# Health utilities and parental quality of life effects for three rare conditions tested in newborns

**DOI:** 10.1186/s41687-019-0093-6

**Published:** 2019-01-22

**Authors:** Norma-Jean Simon, John Richardson, Ayesha Ahmad, Angela Rose, Eve Wittenberg, Brittany D’Cruz, Lisa A. Prosser

**Affiliations:** 1 Ann and Robert H. Lurie Children’s Hospital of Chicago, 225 East Chicago Ave, Chicago, IL 60611 USA; 2 RTI International, 3040 East Cornwallis Road, P.O. Box 12194, Research Triangle Park, NC 27709-2194 USA; 3 Division of Pediatric Genetics, Metabolism and Genomic Medicine, Department of Pediatrics, The University of Michigan Medical School, 4810 Jackson Road, Ann Arbor, MI 48103 USA; 4 Child Health Evaluation and Research Center, Department of Pediatrics, University of Michigan Medical School, 300 North Ingalls Building, Ann Arbor, MI 48109 USA; 5 Harvard TH Chan School of Public Health, 677 Huntington Avenue, Boston, MA 02115 USA; 6 Center for the Evaluation of Value & Risk in Health, Tufts Medical Center, 800 Washington Street, Boston, MA 02111 USA; 7 Health Management and Policy, The University of Michigan School of Public Health, 1415 Washington Heights, Ann Arbor, MI 48109 USA

**Keywords:** Health utilities, Cost-effectiveness, Time trade-off, Pompe, Krabbe, Phenylketonuria, Newborn screening, Spillover, Health disutility, Family effects

## Abstract

**Background:**

Measurement of health utilities is required for economic evaluations. Few studies have evaluated health utilities for rare conditions; even fewer have incorporated disutility that may be experienced by caregivers. This study aimed to (1) estimate health utilities for three rare conditions currently recommended for newborn screening at the state or federal level, and (2) estimate the disutility, or spillover, experienced by parents of patients diagnosed with a rare, heritable disorder.

**Methods:**

A stated-preference survey using a time trade-off approach elicited health utilities for Krabbe disease, phenylketonuria, and Pompe disease at varying stages (mild, moderate, severe) and onset of disease symptoms (infancy, childhood, and adulthood). We recruited respondents from a nationally representative community sample (*n* = 862). Respondents valued disease specific health states in three consecutive question frames: (1) adult health state (> = 18 years of age), (2) child health state (< 18 years of age), and (3) as a parent of a child with a condition (parent spillover state). Corresponding mean utilities were calculated for plausible disease states in adulthood and childhood. Mean disutility was estimated for parental spillover. Predictors of utilities were evaluated using a negative binomial regression model.

**Results:**

More severe conditions and infant health states received lower estimated utility and greater estimated disutility among parents. Conditions with the lowest estimated health utilities were severe infantile Pompe disease (0.40, CI: 0.34–0.46) and infantile Krabbe disease (0.37, CI: 0.32–0.43). Disutility was evident for all conditions evaluated (range: 0.07–0.19).

**Conclusions:**

Rare childhood conditions are associated with substantial estimated losses in quality of life. Evidence of disutility among parents further warrants the inclusion of spillover effects in cost-effectiveness analyses. Continued research is needed to assess and measure the effects of childhood disease from a family perspective.

**Electronic supplementary material:**

The online version of this article (10.1186/s41687-019-0093-6) contains supplementary material, which is available to authorized users.

## Background

Newborn screening has expanded rapidly over the last two decades with the introduction of tandem mass spectrometry, advances in genetic identification, and policy initiatives to improve and align newborn screening resources [1, 2]. Currently, 34 conditions are recommended for screening by the Advisory Committee for Heritable Disorders in Newborns and Children (ACHDNC) [3]. States typically view these recommendations as a minimum set of conditions for which to screen; many states screen for more than 50 conditions [4].

Economic evaluations are conducted to better estimate the implications of expanding newborn screening for recommended disorders. Health utility estimates are required as part of economic evaluation to quantify the burden of disease and derive quality-adjusted life years (QALYs), the outcome measure recommended for use in cost-effectiveness analysis [5–7]. To date few studies have elicited health utilities for conditions recommended for newborn screening; utility estimates are necessary for evaluating whether the averted morbidity and mortality for patients identified through newborn screening are worth the costs associated with earlier detection and treatment of heritable disorders [8].

A growing body of evidence suggests that quality of life effects included in economic evaluations should be considered beyond the individual patient. Parents, family members, and informal caregivers can be significantly affected by the stress and caregiving responsibilities related to a family member’s disease [9–13]. Few studies have directly measured these ‘spillover’ effects. Among studies that have, results vary depending on the disease state, the relationship of a family member to the ill patient, and patient age [14]. Opportunities exist to more comprehensively capture the burden of disease in economic evaluation by considering the quality of life effects on both patients and family members.

**Fig. 1:**
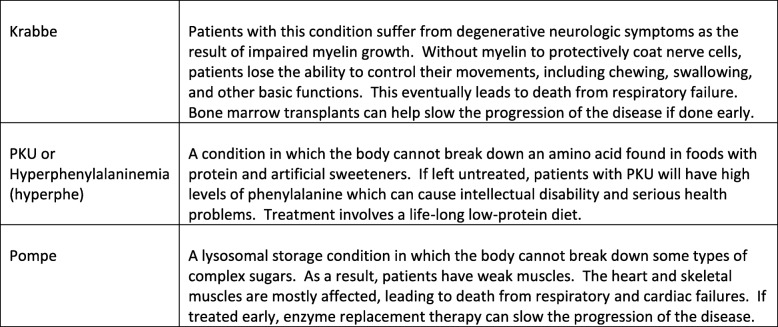
Rare Disorder Descriptions

This study aimed to estimate health utilities to support economic evaluations using a family perspective. We used accepted health utility elicitation techniques to (1) estimate health utilities at varying ages and stages of disease from infancy to adulthood, and (2) estimate parental spillover effects for three rare conditions: Krabbe disease, phenylketonuria (PKU), and Pompe disease (Fig. 1).

These conditions share the substantial risk of death or severe sequelae if not identified and treated close to birth. Additionally, all conditions have either been recommended or nominated by the Advisory Committee for Heritable Disorders in Newborns and Children (ACHDNC) for screening [15–17]. Although Krabbe disease is not currently included on the recommended uniform screening panel endorsed by the ACHDNC, 4 states are currently screening for Krabbe disease with 6 other states in various stages of implementation [18]. Secondary objectives of this study aimed to evaluate differences in health utility valuations based on respondent confidence, predictors of health utility valuations, and use of video health state descriptions.

## Methods

We developed an online, stated-preference survey to elicit health utilities using direct-valuation methods. The time trade-off (TTO) approach asked respondents to trade off a portion of their remaining life to avoid an undesirable health state. The modified time trade off approach utilized in this study has been used previously to elicit utilities for childhood health states [19, 20] and family spillover [11]. Derived utility values are calculated on a scale from (0) dead to (1) perfect health [21].

### Study participants

We invited a nationally representative sample of adults to participate in this study. This community sample was drawn from a commercially available panel of US adults, the GfK KnowledgePanel [22]. GfK recruits panel members via random digit dialing and address-based sampling. Members are compensated for their participation in the panel with personal home internet access. In addition, we identified a convenience sample of adult patients and parents experienced with PKU and mild-hyperphe, a mild variant of PKU, to participate in the survey. Further details regarding the experienced sample recruitment and results can be found in the online appendix (Additional file 1).

### Survey design and development

The survey included four sections: an eligibility screener, TTO practice questions, TTO health state valuations, and questions concerning current wellbeing and socio-demographics. The screener collected information regarding the respondent’s age, gender, and familiarity with the three conditions. Hover-over text was provided to describe the three conditions to identify community members experienced with one of the three conditions.

Health state scenario descriptions for the TTO tasks were developed in consultation with patients and medical experts familiar with Krabbe, PKU, and Pompe disease. Health states were based on a combination of attributes including stage of disease (mild, moderate, or severe), age of symptom onset (infancy, childhood, or adulthood), adherence to therapy (low or high) and treatment. In total, 18 health states were developed for health utility elicitation: 4 PKU, 6 Krabbe, 6 Pompe, and 2 enzyme replacement therapy (ERT) treatment conditions. ERT is a lifelong medical treatment for Pompe disease that slows the progression of disease but is not curative.

**Fig. 2:**
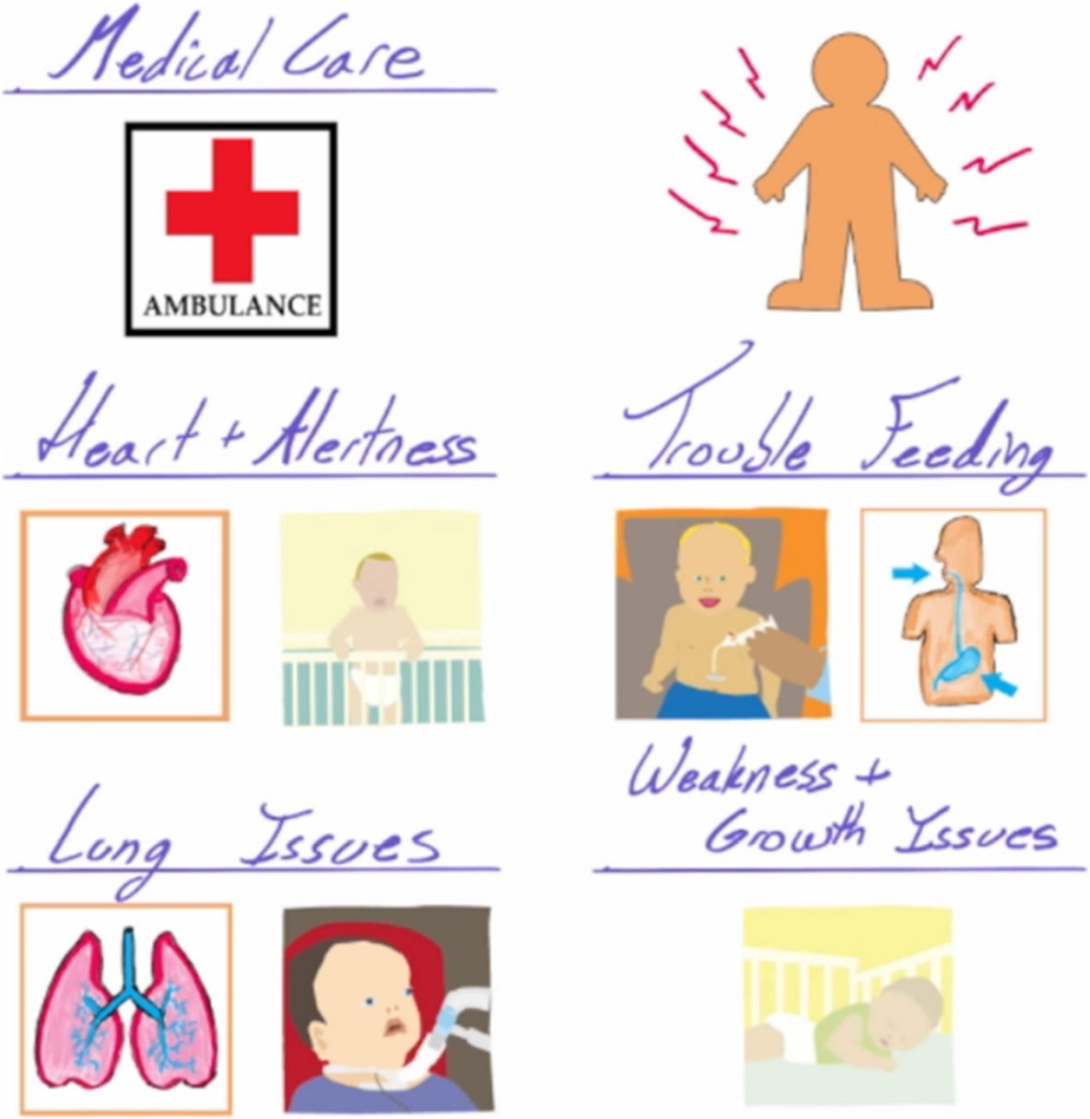
Sample of a Health State Animation*

Health state descriptions included physical, mental, and emotional health domains as well as unique attributes of each disorder like diet and medical care. In addition to written health states, a graphic designer created customized illustrations for a 20–30 s animated video health state. Previous studies have shown that many adults lack basic literacy and numeracy skills required to understand written health states [23, 24]; videos offer an alternative medium to present health states to reduce literacy barriers (Fig. 2). The complete set of health state descriptions is available in the online appendix (Additional file 1).

### TTO health state valuation questions

**Fig. 3:**
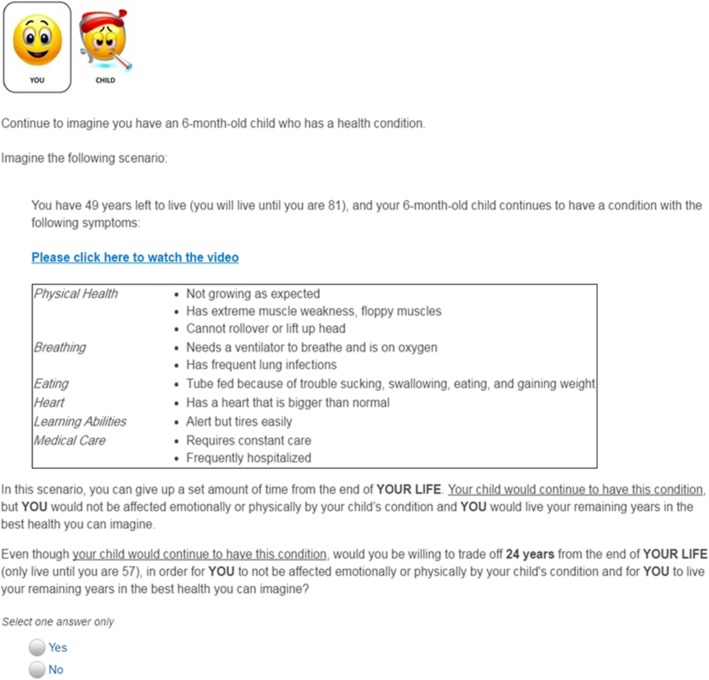
Sample of a Time Trade-Off Question

Respondents completed practice questions to cognitively prepare for the TTO valuation task. After completion of the practice questions, each respondent evaluated 6–9 parallel health states in three successive question frames: (1) adult health state (> = 18 years of age), (2) child health state (< 18 years of age), and (3) as a parent of a child with a condition (parent spillover state). Frame 1 asked the respondent to imagine how *she* would feel if *she* had the hypothetical condition (termed “Adult” frame). The adult frame asked the respondent how many years of *her* life she would trade off to avoid having the condition *herself*. Frame 2 asked the respondent to consider how *her child* would feel if *her child* had the hypothetical condition (termed “Child Patient” frame). The child patient frame question asked the respondent how many years of her life she would tradeoff to avoid *her child* from having the condition. Frame 3 asked the respondent to imagine how *she* would feel if *her child* had the hypothetical condition (termed “Spillover” frame). The spillover frame asked the respondent how many years of *her* life she would trade to avoid *her own* pain and suffering if her child had the condition (Fig. 3). Each respondent received personalized information on remaining life years based on the respondent’s age and national estimates of life expectancy in the United States [25]. After completion of the valuation task, respondents were asked to assess their level of confidence in their responses on a four-point scale: (1) Very confident, (2) Somewhat confident, (3) Not confident, and (4) They were total guesses.

### Socio-demographic questions and metadata

GfK collects socio-demographic information on panel members including race and ethnicity, household income, education, marital status, employment status, and internet access prior to panel participation. We also collected metadata on health state video usage to evaluate any differences in population characteristics and utility ratings among users and non-users.

The survey instrument was tested and refined through cognitive interviews (*n* = 31). The large number of cognitive pretests reflects the complexity of the survey and the valuation task.

### Data collection

The survey was piloted online in February 2016 (*n* = 75). The survey was administered between March and June 2016*;* 2132 individuals were invited to participate, of which 862 individuals completed the survey (40% response rate). Respondents were randomized to complete one of five survey versions (Additional file 1). The survey took an average of 24 min to complete. All survey instruments were approved by the University of Michigan Medical School Institutional Review Board.

### Statistical analysis

Statistical analyses were conducted using STATA v13 statistical software [26]. Primary analyses included summary statistics of the respondent population and mean utility weights for plausible disease health states experienced in adulthood and childhood (TTO Adult Frame and Child Patient Frame). Non-parametric bootstrapping procedures estimated standard errors and confidence intervals around the means. Disutility weights, defined as 1 minus the health state valued, were calculated for the spillover health states (TTO Spillover Frame). In secondary analyses, respondents were dichotomized based on their self-reported confidence in the valuation task. “Confident” respondents included those who were (1) confident or (2) somewhat confident in their valuations; “Not confident” included those respondents who were (3) not confident or responded their answers were (4) total guesses. Summary statistics were re-calculated to evaluate if utility weights differed depending on respondent confidence.

Subsequent analyses included a negative binomial regression model to assess relationships between time trade-off amounts (in years) and respondent characteristics. Responses were normalized by dividing the respondent TTO amounts by the mean TTO amounts for each health state. GEE models were used to adjust for multiple responses per respondent. Evaluated characteristics included video usage, age, sex, race and ethnicity, household income, educational attainment, employment status, marital status, and internet access prior to GfK panel participation. Finally, summary statistics were calculated for respondents using any health state video. Fischer’s exact test evaluated differences in population characteristics among video users and non-users.

## Results

### Respondent characteristics

Respondents (*n* = 862) ranged in age from 18 to 90 years with a median age of 55. Respondent characteristics were similar to the US general population with a few exceptions, namely our sample was older and more likely to be White and of non-Hispanic origin (74%) [27]. Among respondents, 36% held a bachelor’s degree or higher and 61% were married or living with a partner. Approximately 56% of respondents reported being employed at the time of survey participation while 24% reported being retired (Table 1). None of the respondents reported having or knowing someone with PKU, Pompe disease, or Krabbe disease.

**Table 1:** Respondent Characteristics

Characteristic	Community Sample (*n* = 862)	
	Frequency	%
Gender		
Male	446	51.7
Female	416	48.3
Age (years)		
18–24	68	7.9
25–34	116	13.5
35–44	122	14.2
45–54	119	13.2
55–64	211	24.5
≥ 65	226	26.2
Race/Ethnicity		
White, non-Hispanic	635	73.7
Black, non-Hispanic	79	9.2
Other, non-Hispanic	67	7.8
Hispanic	81	9.4
Education		
< 12th Grade, no diploma	60	7.0
High School Graduate	251	29.1
Some college, Associate’s	242	28.1
Bachelor’s Degree or higher	309	35.9
Household Income		
< $25,000	132	15.3
$25,000 - < $50,000	177	20.5
$50,000 - < 75,000	152	17.6
$75,000 - < $100,000	121	14.0
≥ $100,000	280	32.5
Refused	0	–
Respondent Confidence in Valuation Responses^a^		
Confident	717	83.2
Not Confident	139	16.1
Refused	6	0.7

^a^Respondents were dichotomized based on their self-reported confidence in the valuation task. “Confident” respondents included those who were (1) confident or (2) somewhat confident in their valuations; “Not Confident” included respondents who answered (3) not confident or (4) they were total guesses

### Health utility weights

**Fig. 4:**
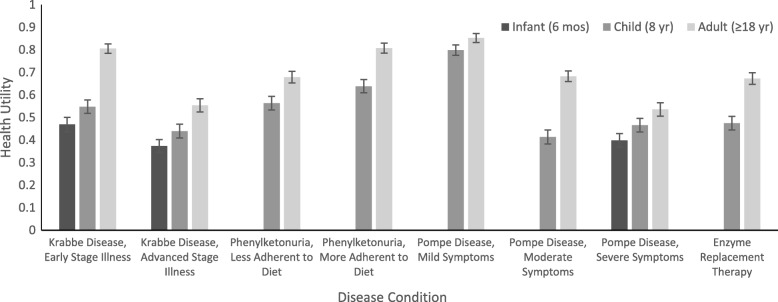
Health Utilities by Disease Condition and Age of Symptom Experience or Onset

Mean health utility weights were calculated for all childhood and adult conditions based on responses to the adult (frame 1) and child (frame 2) TTO questions (Table 2; Fig. 4). Infantile-onset health states elicited the lowest utility across all evaluated conditions. Severe infantile Pompe disease (0.40, CI: 0.34–0.46) and infantile advanced Krabbe disease (0.37, CI: 0.32–0.43) elicited the lowest utilities. Conversely, the highest mean utilities were calculated for milder health states in adulthood. The highest utilities were estimated for adult early stage Krabbe disease (0.81, CI: 0.76–0.85), adult mild Pompe disease (0.85, CI: 0.81–0.89), and adult PKU with high adherence to a low-protein diet (0.81, CI: 0.76–0.85). This trend was also consistent for the enzyme replacement therapy (ERT) treatment condition in which assigned utilities were significantly lower for the childhood treatment states (0.48, CI: 0.42–0.53) compared to the adult treatment states (0.67, CI: 0.62–0.72).

**Table 2:** Health utilities derived from a community sample by disease condition and age of patient

Health State Description	Health Utility		
	N	Mean	95% CI^a^
Krabbe Disease			
Early Stage Illness, 6 months	166	0.469	0.409–0.532
Advanced Stage Illness, 6 months	167	0.374	0.316–0.430
Early Stage Illness, 8 years	168	0.548	0.490–0.606
Advanced Stage Illness,8 yr	168	0.440	0.380–0.500
Early Stage Illness,≥18 yr	167	0.806	0.757–0.847
Advanced Stage Illness,≥18 yr	167	0.554	0.497–0.612
Phenylketonuria			
Less Adherent to Diet, 8 yr	170	0.564	0.506–0.623
More Adherent to Diet,8 yr	171	0.639	0.581–0.696
Less Adherent to Diet, ≥18 yr	171	0.679	0.628–0.730
More Adherent to Diet,≥18 yr	171	0.808	0.762–0.852
Pompe Disease			
Severe Symptoms, 6 months	170	0.399	0.341–0.457
Mild Symptoms, 8 yr	171	0.799	0.750–0.844
Moderate Symptoms, 8 yr	169	0.414	0.355–0.475
Severe Symptoms, 8 yr	169	0.466	0.407–0.525
ERT^b^ Treatment, 8 yr	170	0.475	0.417–0.534
Mild Symptoms, ≥18 yr	170	0.853	0.811–0.892
Moderate Symptoms, ≥18 yr	170	0.683	0.634–0.729
Severe Symptoms, ≥18 yr	171	0.536	0.480–0.594
ERT^b^ Treatment, ≥18 yr	169	0.673	0.621–0.723

Disease health states listed ≥18 yr. of age are derived from Frame 1 TTO: Adult health state questions

Disease health states listed for ages < 18 yr. are derived from Frame 2 TTO: Child health state questions

^a^Bootstrapped

^b^Enzyme replacement therapy

Parent disutility, or spillover, was calculated for all childhood conditions (Table 3). Spillover estimated the burden of disease on a parent of a child with one of the specified conditions. The greater the spillover effect, the greater the quality of life loss to the parent due to the child’s condition. The greatest spillover disutility was assigned to the infantile-onset advanced Krabbe condition (0.19, CI: 0.14–0.24). The lowest spillover disutility was assigned to mild Pompe disease in childhood (0.07, CI: 0.04–0.10). Mean spillover disutility assigned to the PKU conditions, low and high adherence, were similar (0.12 and 0.11 respectively).

**Table 3:** Disutility estimates of parental spillover quantifying health-related quality of life losses due to childhood conditions

	N	Disutility		Spillover decrement relative to child disutility	
		Mean	95% CI^a^	Percent	95% CI
Krabbe Disease					
Early Stage Illness, 6 months	167	0.152	0.110–0.195	32.4	26.9–36.7
Advanced Stage Illness, 6 months	167	0.190	0.141–0.238	50.8	44.6–55.3
Early Stage Illness, 8 years	168	0.136	0.096–0.177	24.8	19.6–29.2
Advanced Stage Illness, 8 yr	168	0.163	0.119–0.208	37.0	31.3–41.6
Phenylketonuria					
Less Adherent to Diet, 8 yr	170	0.120	0.079–0.160	21.3	15.6–25.7
More Adherent to Diet, 8 yr	171	0.110	0.072–0.148	17.2	12.4–21.3
Pompe Disease					
Severe Symptoms, 6 months	170	0.180	0.129–0.230	45.1	37.8–50.3
Mild Symptoms, 8 yr	171	0.072	0.042–0.103	9.0	5.6–12.2
Moderate Symptoms, 8 yr	169	0.162	0.116–0.208	39.1	32.7–43.8
Severe Symptoms, 8 yr	171	0.131	0.090–0.173	28.1	22.1–33.0
ERT Treatment, 8 yr	169	0.155	0.110–0.200	32.6	26.4–37.5

^a^Bootstrapped

### Secondary analyses

Further analyses restricted approximately 16% of respondents who expressed low confidence in their health state valuations. Mean health utility results were similar among the restricted sample compared to the full sample of respondents. Respondents with confidence in their responses generally assigned lower health utility values for more severe conditions in both adulthood and childhood. These differences approached, but did not reach, statistical significance (0.05) except for two conditions, childhood severe Pompe disease (0.43, CI: 0.37–0.50) and the adult ERT treatment condition (0.64, CI: 0.59–0.70; full results not shown).

Results of the regression analysis demonstrated that few respondent characteristics were associated with time trade off valuations. Marital status and employment status were exceptions. Respondents who reported separation from a partner or spouse and respondents temporarily laid-off were less likely to trade off time at the end of their life and therefore assigned higher utilities to described health states compared individuals who were married or employed (Additional file 1).

Finally, descriptive statistics explored the frequency of video use as well as respondent characteristics of video viewers. Videos were most often accessed for health states presented in the first question evaluated; at most 35% of respondents ‘clicked’ on the available video for any given health state; 42% clicked on a video at least once. Video ‘clicks’ progressively decreased as respondents advanced through the survey. Among video users and non-users, significant differences with regard to age and home internet access were observed: video users were more often over 60 years of age and reported having home internet access prior to panel participation (Additional file 1).

## Discussion

This study estimated substantial quality of life losses associated with Pompe disease, PKU, and Krabbe disease. Our results indicate that health utility and spillover effects vary by condition, age of symptom onset, and stage of disease. Trends emerged in which more debilitating conditions and infant onset conditions were associated with lower ratings of utility and higher disutility among parents. Our results corroborate existing research that suggest infantile-onset conditions are more distressing for parents and caregivers compared to conditions experienced in older children, adolescents, or adults [10].

Previous studies have reported health utilities for Pompe disease and sequelae of PKU [28–33], but not Krabbe disease. Studies have typically utilized indirect elicitation methods. Our estimates for adult onset Pompe disease and ERT are similar to other studies [28–30]. In contrast, our health utility estimates for childhood Pompe disease are higher than other estimates [31]. For PKU, our results are consistent with those reported from two studies valuing health related quality of life for mild to moderate developmental delay [32, 33]. Particularly among children, our results also estimate quality of life losses for patient both adherent and less-adherent to a low-protein diet. This is in contrast to other studies which have demonstrated that with early detection of PKU and treatment, quality of life among adolescents and adults is not different than otherwise healthy individuals for studies using patients as respondents [34–36]. This discrepancy is consistent with research that indicates respondents who are inexperienced with a health state (community sample), compared with an experienced patient sample, may overestimate quality of life losses at least for some conditions [37]. When incorporating health utility weights into a cost-effectiveness analysis, it is important to consider the respondent sample. Guidelines for cost-effectiveness analysis typically recommend health utility weights from a community sample for resource allocation decisions [38] and it is important to note that the selection of respondent sample could influence the magnitude of weights.

This study is the first, to our knowledge, to elicit spillover utilities using direct valuation methods for these rare conditions. Parental spillover effects were detected in all of the health conditions evaluated, and were especially notable in severe early infantile and childhood onset conditions. Among our sample, mean disutility or parental spillover estimates ranged from 0.07 for mild Pompe disease in childhood to 0.19 for severe early infantile Krabbe disease. Other studies have reported mean spillover effects within the ranges observed in our study from 0.08 among experienced parents of children with physical limitations [39], 0.10 among parents of children with two rare congenital disorders [40], and mean spillover of 0.26 among parents of children with a severe childhood condition like cancer [11]. Our study is consistent with findings from a previous study utilizing TTO methods in which spillover effects between 0.08–0.27 were estimated for a childhood condition requiring a restricted protein diet [41]. Our findings reiterate the importance of including parental spillover effects into economic evaluation as quality of life losses can be substantial.

In a previous study, we collected preferences for a false positive newborn screening test result [41]. We found that patients assigned a small but significant disutility of 0.003 (0.001 to 0.006) to the false positive newborn screening experience and mean WTP to avoid this experience was $159 (95 to 246). While small at the individual patient level, it is important to consider this value when conducting cost-effectiveness analyses of newborn screening, since false positive rates can vary substantially across target conditions for a given population.

This study has some limitations. First, there are few well-established methods for valuing child health utilities, especially for very young children [14, 42]. A recent review highlighted the lack of evidence for judging the use of direct valuation methods for child health [43]. More research is needed in this area to identify feasible and reliable methods. Additionally, appropriate methods for integrating parent and family spillover remains an active area of discussion. Utilizing the direct elicitation methods employed in this study, it is possible that parental spillover effects are incorporated in child health state valuations, which would result in double counting of the quality of life losses among parents. This is especially possible in the early infantile-onset health states in which it may be hardest for parents to separate the effect of a disease on themselves versus their child. Further understanding and development of methods to measure and incorporate parental spillover can improve cost-effectiveness estimates of childhood interventions.

Second, some challenges are present due to the complexity of the preference-based evaluation task. Wide confidence intervals on many of our estimates indicate substantial variability and uncertainty in measurements. This is a recognized challenge in estimating quality of life for pediatric conditions and is indicative of the complexities of valuing child health for economic evaluation [10, 44]. To mitigate against this complexity, we attempted to find alternative formats to communicate health state descriptions including videos to assist survey respondents in their evaluation of the hypothetical health states. While health utility ratings did not differ based upon video usage, our results indicate that older respondents were more likely to use the videos. Future research should continue to explore a variety of methods to communicate complicated health states to different audiences through available web-based technology.

Third, consistent with conventional approaches to utility elicitation, we did not specify the duration of time spent in a specific health state. This could also have added to the complexity of the questions as respondents may have made assumptions for the duration of time spent in a health state given the severe nature of some of the health states.

Finally, we were limited in selecting an experienced (or patient) sample of respondents due to the rare nature of these conditions. Respondent valuations may not be representative of all experienced adult patients and parents of children with Pompe, PKU or Krabbe disease in the US. As more states begin to screen for these rare diseases, particularly Pompe disease, state-based registries may become more widely available to recruit experienced patients and families to more fully understand the quality of life implications of these rare conditions and their treatment.

## Conclusions

Our results indicate that estimated health utilities for Krabbe disease, phenylketonuria, and Pompe disease vary by condition, age of disease onset, and stage of childhood disease; however, all conditions are associated with substantial quality of life losses for children with these rare conditions and their parents. Parental spillover effects were observed across all childhood conditions in this study and were especially notable among infantile-onset and severe childhood conditions. Finally, quality of life valuations reflect the burden of disease for both patients and parents; our study further supports the inclusion of parental spillover effects in economic evaluations to fully assess the quality of life gains and losses related to early detection and treatment of rare conditions in pediatric populations.

## Additional file

Additional file 1: **Appendix Figure 1.** Health State Descriptions. (DOCX 35 kb)

## Abbreviations

CEA: Cost-effectiveness analysis
ERT: Enzyme replacement therapy
PKU: Phenylketonuria
QALY: Quality-Adjusted Life Year
RUSP: Recommended Uniform Screening Panel
SACHDNC: Secretary’s Advisory Committee for Heritable Disorders in Newborns and Children
TTO: Time-trade-off
