# Supplementary material for: Health utilities and parental quality of life effects for three rare conditions tested in newborns
Source: J Patient Rep Outcomes. 2019 Jan 22;3:4. doi: 10.1186/s41687-019-0093-6 (PMC6342747; doi:10.1186/s41687-019-0093-6)
Supplement: Supplementary file 1 — Appendix Figure 1. Health State Descriptions. (DOCX 35 kb) [file 41687_2019_93_MOESM1_ESM.docx]

Appendix Figure 1. Health State Descriptions

**Krabbe Disease Descriptions**

**Infantile Onset** (6 mos), Early Stage Illness

Physical Health

- Has general muscle weakness, fisted hands, and poor head control
- Normal eyesight and hearing, but sensitive to loud sounds
- Has unexplained fever and seizures
- Difficult to feed and has reflux (vomiting)

Emotional

- Fussier than other babies
- Cries for no apparent reason

Learning Ability

- Not as alert as expected

Medical Care

- Every 3 months has special doctor visits at a major medical center
- Takes medicine to reduce reflux

**Infantile Onset** (6 mos), Advanced Stage Illness

Physical Health

- Poor muscle control and looks very rigid and stiff
- Cannot make coordinated or voluntary body movements
- Frequent seizures
- Unexplained fever
- Unable to swallow food and is tube fed
- Lost nearly all eyesight and hearing
- Has difficulty breathing

Emotional

- Very fussy

Learning Ability

- Is not making any developmental or growth milestones
- Hardly makes any coordinated or voluntary movements

Medical Care

- Every 3 months, has special doctor visits at a major medical center
- Takes medicine to relieve muscle tightness and stiffness and reduce seizures
- End-of-life care focused on comfort is given

**Childhood Onset** (8 yr), Early Stage Illness

Physical Health

- Muscle weakness makes it hard to walk long distances
- Problems with hand coordination
- Minor eyesight and hearing problems
- Able to do self-care tasks like getting dressed, showering, and brushing teeth with little difficulty

Social

- Trouble participating in sports due to muscle strength and coordination issues

Learning Ability

- Mild learning issues have been noticed at school

Medical Care

- Every 3-4 months, go to a doctor at a major medical center for a check-up
- Regularly see physical and occupation therapist

**Childhood Onset** (8 yr), Advanced Stage Illness

Physical Health

- Unable to control and coordinate body movements
- Partially paralyzed and need wheelchair
- Experience frequent seizures
- Poor vision and hearing
- Cannot do self-care tasks like getting dressed, showering, and brushing teeth without help

Social

- Increasingly rely on others
- Difficulty socializing with other children

Learning Ability

- Learning ability has declined and is delayed
- Attends special education classes
- Problems speaking and writing

Medical Care

- Every 3-4 months, go to a doctor at a major medical center for a check-up
- Take medicine to relieve muscle spasms and maintain muscle tone
- Regularly go to physical and occupational therapy

**Adult Onset** (≥18 yr), Early Stage Illness

Physical Health

- Muscle weakness which makes it hard to walk long distances
- Problems with hand coordination
- Experience burning skin sensations in arms or legs
- Minor eyesight and hearing problems
- Able to eat, dress, and brush teeth on own with little difficulty

Social

- Able to maintain a desk job

Learning Ability

- A little trouble with memory and thinking clearly

Medical Care

- Every 3-4 months, go to a doctor at a major medical center for a check-up
- Regularly go to physical and occupational therapy

**Adult Onset** (≥18 yr), Advanced Stage Illness

Physical Health

- Unable to control and coordinate body movements
- Partially paralyzed and need wheelchair
- Experience frequent seizures
- Have lost much of your vision
- Cannot do household chores or self-care tasks like going to the bathroom or showering without help

Social

- Not able to work
- Increasingly rely on others

Learning Ability

- Ability to learn and think clearly has declined
- Problems speaking and writing

Medical Care

- Every 3-4 months, go to a doctor at a major medical center for a check-up
- Take medicine to relieve muscle spasms and maintain muscle tone
- Regularly see a physical and occupational therapist

**Phenylketonuria Descriptions**

**Child** (8 yr), Low Adherence

Diet

- Must be on a strict, low-protein diet
- Avoid high-protein foods like meat, fish, nuts, diary, eggs, and beans.
- Drink special medical formulas
- Eat limited portions of other foods
- Has difficulty following the diet

Learning Ability & Physical Health

- Has learning and behavioral problems
- Is hyperactive (ADHD),
- Problems socializing with other children.

Emotional

- Feels left-out, frustrated, and anxious.

Medical Care

- Talk to a dietitian regularly
- Draw your child’s blood each month
- Once per year, go to a metabolic doctor at a major medical center

**Child** (8 yr), High Adherence

Diet

- Must follow a strict, low-protein diet
- Avoid high-protein foods like meat, fish, nuts, eggs, diary, and beans
- Drink special medical formulas
- Eat a limited portion of other foods
- Does a good job following the diet

Learning Ability & Physical Health

- No learning problems
- Is able to play and keep up with other kids

Emotional

- Can feel left-out and frustrated

Medical Care

- Talk to a dietitian regularly
- Draw your child’s blood each month
- Once per year, go to a metabolic doctor at a major medical center

**Adult** (≥18 yr), Low Adherence

Diet

- Must be on a strict, low-protein diet
- Avoid high-protein foods like meat, fish, nuts, diary, eggs, and beans.
- Drink special medical formulas
- Eat limited portions of other foods
- Spend extra time making and planning meals
- Diet is difficult for you to follow

Learning Ability & Physical Health

- Have hard time learning, thinking clearly, making deadlines and making decisions.
- Have headaches and tremors

Emotional

- Less active, have less energy, and experience mood swings
- Difficult to maintain diet when with friends and family

Medical Care

- Talk to a dietitian regularly
- Draw your blood at home each month
- Once per year, go to a metabolic doctor at a major medical center

**Adult** (≥18 yr), High Adherence

Diet

- Must be on a strict, low-protein diet
- Avoid high-protein foods like meat, fish, nuts, diary, eggs, and beans.
- Drink special medical formulas
- Eat limited portions of other foods
- Spend extra time making and planning meals
- You are able to follow the strict diet

Learning Ability & Physical Health

- Do not have trouble staying physically active
- Participate in activities you enjoy

Emotional

- Difficult to maintain diet when you are with friends and family.

Medical Care

- Talk to a dietitian regularly
- Draw your blood at home each month
- Once per year, go to a metabolic doctor at a major medical center

**Pompe Disease Descriptions**

**Early Infantile Onset,** Severe Symptoms (6 mos)

Physical Health

- Not growing as expected
- Has extreme muscle weakness, floppy muscles
- Cannot rollover or lift up head

Breathing

- Needs a ventilator to breath and is on oxygen
- Has frequent lung infections

Eating

- Tube fed because of trouble sucking, swallowing, eating, and gaining weight

Heart

- Has a heart that is bigger than normal

Learning Ability

- Alert but tires easily

Medical Care

- Requires constant care
- Frequently hospitalized

**Childhood Onset,** Mild Symptoms (8 yr)

Physical Health

- Able to walk without stumbling
- Able to do self-care tasks like showering and getting dressed independently

Breathing

- No breathing problems except during exercise

Eating

- Able to chew and swallow food without difficulty

Learning Ability

- Normal mental development and no learning issues

Social

- Participates in social events and has hobbies

Medical Care

- Visit doctors 4 times a year at a major medical center
- Go to 3 therapists each week for 30-45 minutes

**Childhood Onset,** Moderate Symptoms (8 yr)

Physical Health

- Walk with a waddle and fall often
- Problems climbing stairs or getting up from a chair
- Able to do light household chores and do all self-care tasks like getting dressed and showering
- Tire easily

Breathing

- Problems breathing
- Get frequent headaches and feel daytime sleepiness

Eating

- Trouble chewing, swallowing, eating, and as a result have trouble maintaining a healthy weight

Social

- Not able to participate in sports and other athletic events

Medical Care

- Visit doctors 4 times a year at a major medical center
- Go to 3 therapists each week for 30-45 minutes

**Childhood Onset,** Severe Symptoms (8 yr)

Physical Health

- Needs a wheelchair
- Not able to do self-care tasks like showering and going to the bathroom alone

Breathing

- Needs a ventilator
- Gets frequent headaches
- Feels extreme fatigue and daytime sleepiness

Eating

- Has trouble chewing, swallowing, and as result has problems maintaining weight

Learning Ability

- Has normal mental development and no learning issues

Social

- Has limited hobbies
- Difficulty playing and interacting with other children
- Rely on others for help

Medical Care

- Visits doctors 6-10 times a year at a major medical center
- Goes to 3 therapists each week for 30-45 minutes

**Adult Onset,** Mild Symptoms (≥18 yr)

Physical Health

- Able to walk without stumbling
- Able to bathe, dress, and do most household chores independently

Breathing

- No breathing problems except during exercise

Eating

- Able to chew and swallow food without difficulty

Learning Ability

- No learning issues

Social

- Work at a job that does not require lifting or bending
- Participates in social events and hobbies

Medical Care

- Visit doctors 4 times a year at a major medical center
- Go to 3 therapists each week for 30-45 minutes

**Adult Onset,** Moderate Symptoms (≥18 yr)

Physical Health

- Walk with a waddle and fall often
- Problems climbing stairs or getting up from a chair
- Able to do light household chores and do all self-care tasks like getting dressed and showering
- Tire easily

Breathing

- Problems breathing
- Get frequent headaches and feel daytime sleepiness

Eating

- Trouble chewing, swallowing, eating, and as a result have trouble maintaining a healthy weight

Social

- No longer participate in sports and other athletic events

Medical Care

- Visit doctors 4 times a year at a major medical center
- Go to 3 therapists each week for 30-45 minutes

**Adult Onset,** Severe Symptoms (≥18 yr)

Physical Health

- Need a wheelchair
- Not able to do self-care tasks like showering and going to the bathroom alone

Breathing

- Need a ventilator to breathe
- Get frequent headaches
- Experience extreme fatigue
- Shortness of breath when speaking

Eating

- Trouble chewing, swallowing and eating. As a result it is hard to maintain and gain weight

Learning Ability

- No learning issues

Social

- Difficult to work and do household chores
- Rely on others for help
- Have lost contact with some friends

Medical Care

- Visit doctors 6-10 times a year at a major medical center
- Go to 3 therapists each week for 30-45 minutes

**Enzyme Replacement Therapy**

Treatment

- Every 1-2 weeks at home or nearby hospital
- Lasts 6-8 hours, including travel and wait time
- Treatment is given through and IV (infusion) into the bloodstream
- Requires being hooked up to a machine
- Sometimes experience allergic reactions and can include fever, trouble breathing, rashes, nausea, tremors, and restlessness

Appendix Figure. 2

| **Survey Version** | **Question Type & Assigned Health State** | | | | | | |
| --- | --- | --- | --- | --- | --- | --- | --- |
| Block | Set | You -Adult  (T1) | Child (T2) | Spillover (T3) | You-Adult  (E1) | Child (E2) | Spillover (E3) |
|  |  | Disease Health States | | | ERT Treatment Health States | | |
| PKU | 1 | 3 | 4 | 4 |  |  |  |
|  | 2 | 1 | 2 | 2 |  |  |  |
| Pompe | 1 | 5* | 6 | 6 |  |  |  |
|  | 2 | 7 | 8 | 8 |  |  |  |
|  | 3 | 9 | 10 | 10 |  |  |  |
| Pompe | 1 | 12 | 13* | 13* | 22 | 22 | 22 |
| Krabbe | 1 | 14* | 15 | 15 |  |  |  |
|  | 2 | 16* | 17 | 17 |  |  |  |
| Krabbe | 1 | 18 | 19 | 19 |  |  |  |
|  | 2 | 20 | 21 | 21 |  |  |  |

* Denotes a health state is not considered plausible and therefore were not used to calculate health utility weights and are not described in the results.

Health States by Number:

| 1 | PKU - More Adherent to Diet,≥18 yr |
| --- | --- |
| 2 | More Adherent to Diet,8 yr |
| 3 | Less Adherent to Diet, ≥18 yr |
| 4 | PKU- Less Adherent to Diet, 8 yr |
| 5* | Pompe – Adult Severe |
| 6 | Pompe - Severe Symptoms, 6 months |
| 7 | Pompe - Mild Symptoms, ≥18 yr |
| 8 | Pompe - Mild Symptoms, 8 yr |
| 9 | Pompe -Severe Symptoms, ≥18 yr |
| 10 | Pompe - Severe Symptoms, 8 yr |
| 12 | Pompe - Moderate Symptoms, ≥18 yr |
| 13* | Pompe - Moderate Symptoms, 8 yr |
| 14* | Krabbe – Adult Early Stages |
| 15 | Krabbe - Early Stage Illness, 6 months |
| 16* | Krabbe – Adult Advanced |
| 17 | Advanced Stage Illness, 6 months |
| 18 | Krabbe - Early Stage Illness,≥18 yr |
| 19 | Krabbe - Early Stage Illness, 8 years |
| 20 | Krabbe - Advanced Stage Illness,≥18 yr |
| 21 | Krabbe - Advanced Stage Illness,8 yr |
| 22 | ERT^b^ Treatment |

b. Enzyme replacement therapy

| Appendix Table 1. Negative binomial regression model predicting normalized time tradeoff values. Risk ratios, confidence intervals and p-values.* | | | | |
| --- | --- | --- | --- | --- |
| **Covariates** | **Risk Ratio** | **95% Confidence Interval** | | **P-Value** |
| **Clicked on video link** |  |  |  |  |
| No | -- | -- | | -- |
| Yes | 1.06 | 0.92 - 1.22 | | 0.454 |
| **Age** | 0.88 | 0.74 - 1.04 | | 0.137 |
| **Years remaining†** | 0.88 | 0.73 - 1.06 | | 0.186 |
| **Age x Years remaining** | 1 | 1 - 1 | | 0.697 |
| **Sex** |  |  |  |  |
| Male | -- | -- | | -- |
| Female | 1.01 | 0.88 - 1.17 | | 0.868 |
| **Race and ethnicity** |  |  |  |  |
| White, Non-Hispanic | -- | -- | | -- |
| Black, Non-Hispanic | 0.82 | 0.60 - 1.12 | | 0.212 |
| Other, Non-Hispanic | 0.86 | 0.62 - 1.20 | | 0.371 |
| Hispanic | 1.01 | 0.77 - 1.33 | | 0.927 |
| 2+ Races, Non-Hispanic | 1.17 | 0.78 - 1.77 | | 0.451 |
| **Household income** |  |  |  |  |
| $0 to $49,999 | -- | -- | | -- |
| $50,000 to $99,999 | 0.89 | 0.73 - 1.08 | | 0.223 |
| $100,000 to $149,999 | 0.86 | 0.70 - 1.04 | | 0.124 |
| $150,000 + | 0.83 | 0.65 - 1.05 | | 0.12 |
| **Education** |  |  |  |  |
| 5th to 12th grade, no diploma | -- | -- | | -- |
| High school graduate | 1.02 | 0.72 - 1.45 | | 0.897 |
| Some college, no degree | 1 | 0.68 - 1.47 | | 0.993 |
| Associate degree | 1.14 | 0.76 - 1.72 | | 0.515 |
| Bachelor's degree | 0.91 | 0.63 - 1.33 | | 0.636 |
| Master's degree | 0.89 | 0.59 - 1.33 | | 0.559 |
| Professional or Doctorate degree | 0.83 | 0.49 - 1.44 | | 0.513 |
| **Marital status** |  |  |  |  |
| Married | -- | -- | | -- |
| Widowed | 0.94 | 0.65 - 1.37 | | 0.761 |
| Divorced | 1.11 | 0.88 - 1.40 | | 0.376 |
| Separated | 0.42 | 0.24 - 0.74 | | 0.003‡ |
| Never married | 1.08 | 0.87 - 1.35 | | 0.47 |
| Living with partner | 0.91 | 0.70 - 1.19 | | 0.497 |
| **Current employment** |  |  |  |  |
| Working, as a paid employee | -- | -- | | -- |
| Working, self-employed | 0.9 | 0.68 - 1.18 | | 0.44 |
| Not working, on temporary layoff from a job | 0.23 | 0.09 - 0.58 | | 0.002‡ |
| Not working, looking for work | 0.78 | 0.51 - 1.21 | | 0.272 |
| Not working, retired | 0.86 | 0.69 - 1.08 | | 0.195 |
| Not working, disabled | 0.91 | 0.68 - 1.23 | | 0.551 |
| Not working, other | 0.84 | 0.61 - 1.14 | | 0.257 |
| **Home internet access** |  |  |  |  |
| No | -- | -- | | -- |
| Yes | 1.09 | 0.85 - 1.41 | | 0.492 |
| Note: N=5,071. "--" represents the reference group for categorical variables. The responses were normalized by dividing the time trade-off values by the mean time trade-off for the health state. Responses were clustered by individual.  *Pseudo--R^2^ = 0.0255  †Expected years remaining based on U.S. Life Tables  ‡p<0.05 | | | | |

| Appendix Table 2. Characteristics of those that used the videos and those that did not use the videos | | | | | | | | |
| --- | --- | --- | --- | --- | --- | --- | --- | --- |
| **Demographics** | **No Video Use** | | | **Any Video Use** | | | **P-Value** |  |
|  | **Frequency** | **Percent** | | **Frequency** | **Percent** | |  |  |
|  | **(n=503)** |  |  | **(n=362)** |  |  |  |  |
| **Sex** |  |  | |  | |  |  |  |
| Male | 265 | 52.68% | | 182 | 50.28% | | 0.491 |  |
| Female | 238 | 47.32% | | 180 | 49.72% | |  |  |
| **Age** |  |  | |  | |  |  |  |
| 18 to 29 | 87 | 17.30% | | 45 | 12.43% | | 0.002* |  |
| 30 to 44 | 114 | 22.66% | | 63 | 17.40% | |  |  |
| 45 to 59 | 140 | 27.83% | | 94 | 25.97% | |  |  |
| 60+ | 162 | 32.21% | | 160 | 44.20% | |  |  |
| **Race and ethnicity** |  |  | |  | |  |  |  |
| White, non-Hispanic | 369 | 73.36% | | 267 | 73.76% | | 0.668 |  |
| Black, non-Hispanic | 50 | 9.94% | | 30 | 8.20% | |  |  |
| Other, non-Hispanic | 29 | 5.77% | | 17 | 4.70% | |  |  |
| Hispanic | 42 | 8.35% | | 39 | 10.77% | |  |  |
| 2+ races, non-Hispanic | 13 | 2.58% | | 9 | 2.49% | |  |  |
| **Household income** |  | |  |  |  | |  |  |
| $0 to $49,999 | 188 | 37.38% | | 121 | 33.43% | | 0.497 |  |
| $50,000 to $99,999 | 162 | 32.21% | | 114 | 31.49% | |  |  |
| $100,000 to $149,999 | 100 | 19.88% | | 83 | 22.93% | |  |  |
| $150,000 + | 53 | 10.54% | | 44 | 12.15% | |  |  |
| **Education** |  | |  |  |  | |  |  |
| 5th to 12th grade, no diploma | 38 | 7.55% | | 22 | 6.08% | | 0.488 |  |
| High school graduate | 157 | 31.21% | | 95 | 26.24% | |  |  |
| Some college, no degree | 94 | 18.69% | | 65 | 17.96% | |  |  |
| Associate degree | 47 | 9.34% | | 36 | 9.94% | |  |  |
| Bachelor's degree | 93 | 18.49% | | 81 | 22.38% | |  |  |
| Master's degree | 57 | 11.33% | | 46 | 12.71% | |  |  |
| Professional or Doctorate degree | 17 | 3.38% | | 17 | 4.70% | |  |  |
| **Marital Status** |  | |  |  |  | |  |  |
| Married | 277 | 55.07% | | 224 | 61.88% | | 0.091 |  |
| Widowed | 33 | 6.56% | | 19 | 5.25% | |  |  |
| Divorces | 43 | 8.55% | | 41 | 11.33% | |  |  |
| Separated | 9 | 1.79% | | 4 | 1.10% | |  |  |
| Never Married | 123 | 24.45% | | 64 | 17.68% | |  |  |
| Living with partner | 18 | 3.58% | | 10 | 2.76% | |  |  |
| **Current employment** |  | |  |  |  | |  |  |
| Working - as a paid employee | 262 | 52.09% | | 163 | 45.03% | | 0.329 |  |
| Working - self-employed | 38 | 7.55% | | 22 | 6.08% | |  |  |
| Not working - on temporary layoff | 2 | 0.40% | | 3 | 0.83% | |  |  |
| Not working - looking for work | 21 | 4.17% | | 19 | 5.25% | |  |  |
| Not working - retired | 110 | 21.87% | | 96 | 26.52% | |  |  |
| Not working - disabled | 32 | 6.36% | | 28 | 7.73% | |  |  |
| Not working - other | 38 | 7.55% | | 31 | 8.56% | |  |  |
| **Home Internet Access** |  |  | |  |  | |  |  |
| No | 90 | 17.89% | | 40 | 11.05% | | 0.007* |  |
| Yes | 413 | 82.11% | | 322 | 88.95% | |  |  |

**Additional appendix material**

Estimated health utilities and spillover effects in respondents experienced with PKU or mild-hyperphe (n=67)

A small convenience sample of adult patients and adult parents/caregivers were identified to participate in this study through a state newborn screening registry. This sample was included in the study to determine if health valuations differed between the general populations and respondents with lived experiences.

Experienced individuals were asked to participate in the study if they were (1) 18 years of age or older and diagnosed with either PKU or mild-hyperphe; or (2) a parent or guardian of a child (<18 years of age), diagnosed with PKU or mild-hyperphe. Mild-hyperphe is a less severe form or PKU and participants familiar with this disorder were therefore included as having experience or first-hand knowledge of the condition. Eligible participants received a letter from a cooperating state health department explaining the study via postal mail. Participants accessed the survey using a web-link enclosed in the letter and received a $25.00 debit card for participating.

Similar in methodology to the community sample, the experienced sample first completed practice time-trade-off questions in order to prepare for the valuation task. In addition, the adult PKU or mild-hyperphe patients completed two adult patient PKU health state questions (Frame 1). Experienced parents or guardians completed an additional set of child health sates (frame 2), and parental spillover (frame 3).

**Results**

Experienced respondents (n=67) ranged in age from 18 - 64 years with a median age of 34 and were predominately white, non-Hispanic (94%). Of the experienced sample, 67% were adults diagnosed with PKU or mild-hyperphe (n=45), and 33% identified as a parent or caregiver of a child with PKU or mild-hyperphe (n=22). Approximately 46% had attained a bachelor's degree or higher (Table 1).

Tables X and XX detail the health utilities and disutility elicited from the experienced sample. Generally, adults experienced with PKU evaluated health states at higher mean utility and lower disutility compared to our inexperienced community sample in our study. These results suggest that inexperienced, community samples may overestimate quality of life losses.

| Appendix Table 3. Experienced sample respondent Characteristics | | | | | |
| --- | --- | --- | --- | --- | --- |
|  | Experienced Sample,  PKU Adult  (n=45) | | Experienced Sample,  PKU Parent  (n=22) | | |
| **Characteristic** | **Frequency** | **%** | **Frequency** | **%** |  |
| **Gender** |  | |  | | |
| Male | 12 | 26.7 | 2 | 9.1 |  |
| Female | 33 | 73.3 | 20 | 90.9 |  |
| **Age (years)** |  |  |  |  |  |
| 18-24 | 11 | 24.4 | 0 | – |  |
| 25-34 | 17 | 37.8 | 6 | 27.3 |  |
| 35-44 | 7 | 15.6 | 11 | 50.0 |  |
| 45-54 | 8 | 17.8 | 3 | 13.6 |  |
| 55-64 | 2 | 4.4 | 2 | 9.1 |  |
| ≥65 | 0 | – | 0 | – |  |
| **Race/Ethnicity** |  | |  | | |
| White, non-Hispanic | 41 | 91.1 | 22 | 100.0 |  |
| Black, non-Hispanic | 0 | – | 0 | – |  |
| Other, non-Hispanic | 3 | 6.6 | 0 | – |  |
| Hispanic | 1 | 2.2 | 0 | – |  |
| **Education** |  | |  | | |
| < 12^th^ Grade, no diploma | 1 | 2.2 | 0 | – |  |
| High School Graduate | 12 | 26.7 | 1 | 4.5 |  |
| Some college, Associate’s | 14 | 31.1 | 8 | 36.4 |  |
| Bachelor’s Degree or higher | 18 | 40.0 | 13 | 59.1 |  |
| **Household Income** |  | |  | | |
| < $25,000 | 14 | 31.1 | 1 | 4.5 |  |
| $25,000 - < $50,000 | 13 | 29.0 | 7 | 31.8 |  |
| $50,000 - < 75,000 | 8 | 17.8 | 4 | 18.2 |  |
| $75,000 - <$100,000 | 2 | 4.4 | 6 | 13.3 |  |
| ≥$100,000 | 10 | 22.2 | 5 | 22.7 |  |
| Refused | 1 | 2.2 | 0 | – |  |
| **Respondent Confidence in Valuation Responses^a^** |  | |  | | |
| Confident | 41 | 91.1 | 22 | 100.0 |  |
| Not Confident | 4 | 8.9 | 0 | – |  |
| Refused | 0 | – | 0 | – |  |
| a. Respondents were dichotomized based on their self-reported confidence in the valuation task. “Confident” respondents included those who were (1) confident or (2) somewhat confident in their valuations; “Low Confidence” included respondents who answered (3) not confident or (4) they were total guesses. | | | | | |

| Appendix Table 4. Health utilities derived from the experienced sample by disease condition and age of patient (Frame 1 and Frame 2). | | | |
| --- | --- | --- | --- |
| **Health State Description** | Health Utility | | |
|  | **N** | **Mean** | **95% CI** |
| **Phenylketonuria**  Less Adherent to Diet, 8 yr^a,b^ | 22 | 0.569 | 0.390 - 0.739 |
| More Adherent to Diet, 8 yr^a,b^ | 22 | 0.622 | 0.441 - 0.792 |
| Less Adherent to Diet, ≥18 yr | 45 | 0.812 | 0.737 - 0.878 |
| More Adherent to Diet, ≥18 yr | 45 | 0.916 | 0.857 - 0.965 |
| a. Bootstrapped  b. Parent proxies as caregivers of children with PKU | | | |

| Appendix Table 5. Disutility estimates of parental spillover due to caring for a child with varying adherence to a prescribed a low-protein PKU diet. | | | |  |
| --- | --- | --- | --- | --- |
|  | **N** | **Mean** | **95% CI^a^** |  |
| **Experienced Parent with Child** |  |  |  |  |
| Less Adherent to Diet | 22 | 0.096 | 0.005 - 0.221 |  |
| More Adherent to Diet | 22 | 0.021 | 0.001 - 0.050 |  |
| ^a^Bootstrapped | | | | |
